# Autism likelihood in infants born to mothers with asthma is associated with blood inflammatory gene biomarkers in pregnancy

**DOI:** 10.1016/j.bbih.2024.100845

**Published:** 2024-08-13

**Authors:** Vanessa E. Murphy, Olivia M. Whalen, Evan J. Williams, Peter G. Gibson, Linda E. Campbell, Frini Karayanidis, Carly A. Mallise, Alix Woolard, Annelies L. Robijn, Joerg Mattes, Adam M. Collison, Alison E. Lane, Katherine J. Baines

**Affiliations:** a School of Medicine and Public Health, College of Health, Medicine and Wellbeing, The University of Newcastle, Callaghan, NSW, 2308, Australia; b School of Psychological Sciences, College of Engineering, Science and Environment, The University of Newcastle, Callaghan, NSW, 2308, Australia; c School of Biomedical Sciences and Pharmacy, College of Health, Medicine and Wellbeing, The University of Newcastle, Callaghan, NSW, 2308, Australia; d Department of Respiratory and Sleep Medicine, John Hunter Hospital, Newcastle, NSW, 2305, Australia; e Population Health, Hunter New England Local Health District, Wallsend, NSW, 2287, Australia; f Telethon Kids Institute, Perth Children's Hospital, Perth, WA, 6009, Australia; g Paediatric Respiratory and Sleep Medicine Department, John Hunter Children's Hospital, Newcastle, NSW, 2305, Australia; h Olga Tennison Autism Research Centre, La Trobe University, Melbourne, VIC, 3086, Australia

**Keywords:** Inflammation, Gene expression, Offspring, Sensory regulation, Social communication, Biomarkers

## Abstract

Mothers with asthma or atopy have a higher likelihood of having autistic children, with maternal immune activation in pregnancy implicated as a mechanism. This study aimed to determine, in a prospective cohort of mothers with asthma and their infants, whether inflammatory gene expression in pregnancy is associated with likelihood of future autism.

Mothers with asthma were recruited to the Breathing for Life Trial. RNA was extracted from blood samples collected at mid-pregnancy. 300 ng total RNA was hybridized with the nCounter Human Inflammation gene expression panel (Nanostring Technologies, 249 inflammation-related genes). Parents completed the First Year Inventory (FYI) at 12-month follow-up, which assessed an infant's likelihood for autism across 2 behavioural domains: social communication and sensory regulation. A total score ≥19.2 indicated increased likelihood for future autism.

Inflammatory gene expression was profiled from 24 mothers: four infants scored in the high autism likelihood range; 20 scored in the low autism likelihood range. Six inflammatory genes were differentially expressed and significantly up-regulated in the high autism likelihood group: *CYSLTR2*, *NOX1*, *C1QA*, *CXCL10*, *C8A*, *IL23R*. mRNA count significantly correlated with social communication FYI score for *CYSLTR2* (Pearson r = 0.46, p = 0.024) and *CXCL10* (r = 0.43, p = 0.036) and with sensory regulation score for *ALOX5* (r = −0.43, p = 0.038) and *MAFK* (r = −0.46, p = 0.022).

In this proof-of-concept study, inflammatory gene expression during pregnancy in mothers with asthma was associated with an infant's likelihood of future autism as well as scores relating to social communication and sensory regulation.

## 1 Introduction

Autism, a persistent neurodevelopmental condition, is characterised by differences in social communication and interaction, and the presence of restricted, repetitive behaviours and interests. Autism is heterogeneous, in that individuals present with different characteristics and needs, requiring personalised support strategies. Maternal immune activation (MIA) has been implicated as a potential mechanism in the emergence of neurodevelopmental conditions such as autism in infants (Han et al., 2021; Jain et al., 2021). Changes in the intrauterine environment, including alterations in immune mediators which cross the placenta, may affect fetal neurogenesis and the complex processes involved in brain development.

Asthma is an inflammatory disease of the airways, and the leading immune disease to complicate pregnancy, occurring in 8–12% of pregnancies worldwide (Sawicki et al., 2011; Hansen et al., 2013; Rejno et al., 2014). We published the first systematic review examining the relationship between maternal asthma and offspring cognitive and behavioural outcomes. Four of the ten studies showed an increased likelihood of autism or intellectual disability in children born to mothers with asthma (Whalen et al., 2019). However, the studies had weak designs and none investigated potential mechanisms linking MIA and autism. Since then, additional studies have shown that children born to mothers with asthma are at increased odds of autism (Croen et al., 2019; Gong et al., 2019). A 2024 publication, of 311 autistic children and 967 from the general population in California, found that the odds of autism in children whose mothers had asthma was 1.62 (95% CI 1.15–2.29) (Croen et al., 2024).

In addition, there is emerging evidence that MIA may also influence phenotypic expression of autism. Specifically, autistic children whose mothers had immune conditions were more likely to experience increased behavioural and emotional difficulties (Patel et al., 2020). Another study reported more severe social difficulties in autistic children whose mothers had a history of allergies and asthma, compared to autistic children whose mothers had no chronic immune activation history (Patel et al., 2018).

Few studies have examined relationships between maternal blood biomarkers during pregnancy and offspring developmental outcomes. Two such studies examined mid-pregnancy maternal serum cytokine levels (Goines et al., 2011; Carter et al., 2021), and both identified alterations in serum interleukin (IL)-4 in mothers whose children go on to have autism diagnosed in early life. Goines et al. showed that having elevated IL-4 (as well as elevated interferon [IFN]-g and IL-5) in maternal serum at 15–19 weeks gestation, was associated with a greater likelihood of autism in the offspring by age 5 years. On the other hand, Carter et al. showed significantly lower levels of serum IL-4 at 20 weeks gestation in mothers with autistic children, compared to mothers without autistic children.

With asthma occurring commonly in pregnancy and the increasing prevalence of autism, research into associations between maternal asthma and inflammation in pregnancy and autism phenotypes in early life is warranted. In this proof-of-concept study, we aimed to investigate whether inflammatory gene biomarkers in maternal blood were associated with an increased likelihood of autism in infancy.

## 2 Materials and methods

Participants: Participants were pregnant women recruited as part of the Breathing for Life Trial (Murphy et al., 2016), a parallel group randomized controlled trial of a novel asthma management strategy vs usual care (Australian New Zealand Clinical Trials Registry- 12613000202763), and their infants who participated in the prospective Breathing for Life Trial- Infant Development follow-up study (Mallise et al., 2021; Woolard et al., 2023; Whalen et al., 2024). All participants gave written informed consent before participation. Ethics approval was obtained from the Hunter New England Local Health District Human Research Ethics Committee (reference numbers 12/10/17/3.04 main trial, 15/05/20/4.05 infant developmental follow-up). Pregnant women were recruited between 12- and 23-weeks’ gestation from the antenatal clinic of John Hunter Hospital, Newcastle. Baseline data collection included maternal age, body mass index (BMI, estimated from height and weight), gestational age, self-reported smoking status, current asthma symptoms and medication use. Lung function was measured by spirometry (EasyOne Spirometer, NicheMedical North Sydney, Australia) and asthma control was characterised as well controlled, partly controlled or uncontrolled according to the Global Initiative for Asthma (GINA) criteria, using asthma symptoms and short acting beta_2_-agonist (SABA) use in the previous week.

At 12 months of age, we assessed the infant's likelihood for autism using the First Year Inventory (FYI 2.0) (Reznick et al., 2007), a validated, parent-report tool which identifies early signs of autism and neurodevelopmental disability in two behavioural domains: social communication and sensory regulation. A total FYI score of ≥19.2 was used to identify infants with high likelihood of autism, as 44% of babies meeting this criteria are likely to go on to be diagnosed with autism at three years of age (Turner-Brown et al., 2013). In addition, 85% of infants meeting both FYI thresholds of ≥22.5 in the social communication domain, and ≥14.75 in the sensory regulatory domain are likely to experience developmental concerns (autism and non-autism) by three years of age (Turner-Brown et al., 2013).

Sample collection and analysis: Peripheral non-fasting blood samples were collected at the baseline pregnancy visit (prior to randomisation to the novel asthma management strategy) in PAXgene tubes (BD, Franklin Lakes, New Jersey, USA) as per the manufacturer's instructions.

RNA was extracted using the PAXgene Blood RNA Kit (Qiagen) according to the manufacturer's instructions, using automated extraction with the QIAcube (Qiagen, Hilden, Germany). Isolated total RNA samples were assayed for quality (Agilent 2100 Bioanalyser, Agilent Technologies, Santa Clara, CA, USA) and quantity (Quant-iT RiboGreen, Life Technologies, Carlsbad, CA, USA).

Data analysis: Blood inflammatory gene expression was measured from the extracted RNA samples using the nCounter Human Inflammation V2 gene expression panel (Nanostring Technologies, Seattle, WA, USA). Briefly, 300 ng of total RNA was hybridized with the inflammatory code set, at 65ᵒC for 16 h. The hybridized RNA was placed on the automated prep station and transferred to the sample cartridge. Finally, the cartridge was scanned on the nCounter and the files exported into nSolver analysis software for further analyses. Detectable genes in a sample were defined as having a count >20 mRNA molecules.

Quality Control checks (binding density and image quality) were run on the raw data and the background threshold was set for all sample counts using the geometric mean of the negative controls. Data was normalized to the geometric mean of all positive controls, and the housekeeping genes CLTC and GAPDH that demonstrated the best stability values in this dataset (Lindbjerg Andersen et al., 2004). In this sample, no values fell outside the range of detection. To determine the differentially expressed genes between autism likelihood groups, the Welch-Satterthwaite *t*-test was performed in nSolver on normalized counts. Fold changes were calculated as ratios between the geometric means of the respective sample groups. All expression data was represented as normalized counts exported from nSolver.

The Search Tool for the Retrieval of Interacting Genes (STRING: http://string-db.org/) database v11(Szklarczyk et al., 2019) was used to investigate protein-protein interactions between differentially expressed genes. A minimum required interaction score of medium confidence (>0.4) was applied and only proteins matching the differentially expressed gene list (first shell query proteins) were included in the analysis for maximum number of interactions. The network edges were marked as confidence where the line thickness indicates the strength of the supporting data. Disconnected nodes were removed.

Statistical analyses on normalized counts exported from nSolver and clinical data were performed using Stata version 17.0 (StataCorp, College Station, TX) or GraphPad Prism Software version 7.04 (GraphPad Software LLC, La Jolla, CA). Normality of the data was assessed using the D'Agostino-Pearson Normality test. Differences between autism likelihood groups were assessed using the unpaired two-tailed t–test (or Mann-Whitney *t*-test for non-parametric data). Parametric and non-parametric data are represented as mean ± SD or median (interquartile range) respectively. For frequency data, the Fisher's exact test was used. For correlations, the Pearson's correlation coefficient test was used. A p-value of <0.05 was considered significant.

## 3 Results

Of the 731 mothers with asthma who participated in the Breathing for Life Trial at the Newcastle site (Murphy et al., 2016, 2022), 122 infants completed a developmental assessment at 12 months of age, and 91 completed the FYI questionnaire. Of these, matching maternal mid-pregnancy blood samples were available from 24 mothers. Based on the total FYI score, four infants (2 male, 2 female) were determined to have an increased likelihood of autism (“high likelihood” group, mean FYI score 25.4 ± 1.7 SD, range 23–27), leaving 20 infants (11 male, 9 female) with low autism likelihood (“low likelihood” group, mean FYI score 9.6 ± 5.3 SD, range 0–19, p < 0.01). The four infants at high likelihood also met the threshold within the FYI social communication domain, with scores in this domain significantly higher in the high likelihood group (mean 29.4 ± 4.8 SD, range 23–35) than domain scores for the low likelihood group (mean 6.8 ± 7.1 SD, range 0–19, p < 0.01).

There were no significant differences between high and low autism likelihood groups in terms of maternal age, BMI, smoking, parity or gestational age at sample collection, or characteristics of maternal asthma during pregnancy, including lung function, asthma control and randomisation group (data not shown).

**Fig. 1:**
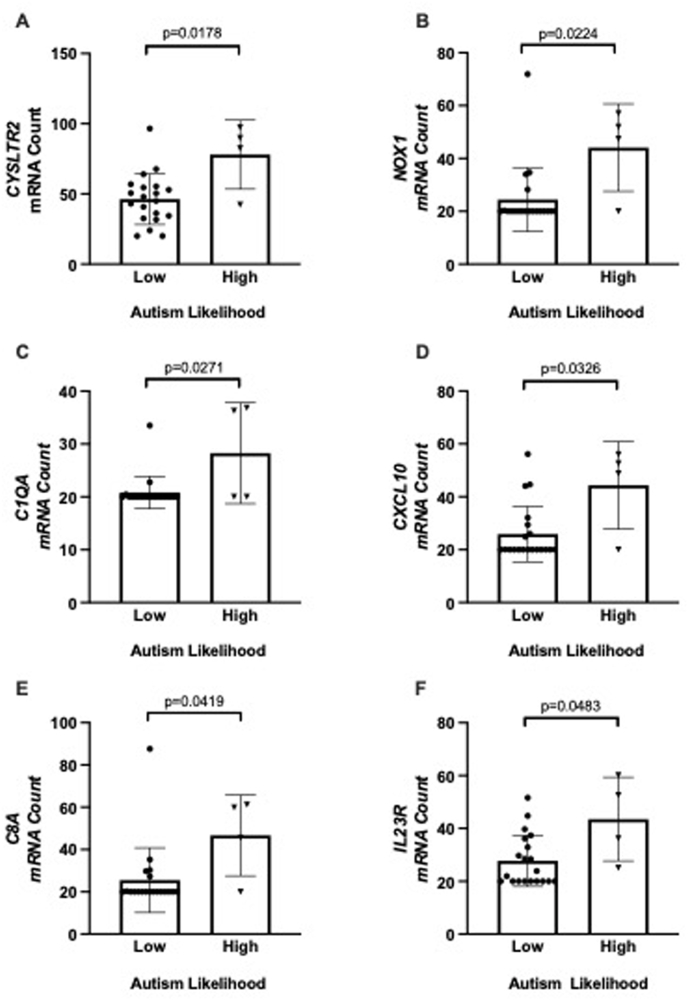
Differentially expressed genes associated with autism likelihood and social communication domain score. Graphs show individual data points, with the bar representing mean, and error bars the standard deviation.

Six genes were significantly differentially expressed between autism likelihood (social communication) groups: *CYSLTR2, NOX1, C1QA, CXCL10, C8A, IL23R* (all up-regulated, p < 0.05, Fig. 1). There were significant correlations between mRNA count and social communication domain score for *CYSLTR2* (Pearson r = 0.46, p = 0.023), and *CXCL10* (Pearson r = 0.43, p = 0.038).

**Fig. 2:**
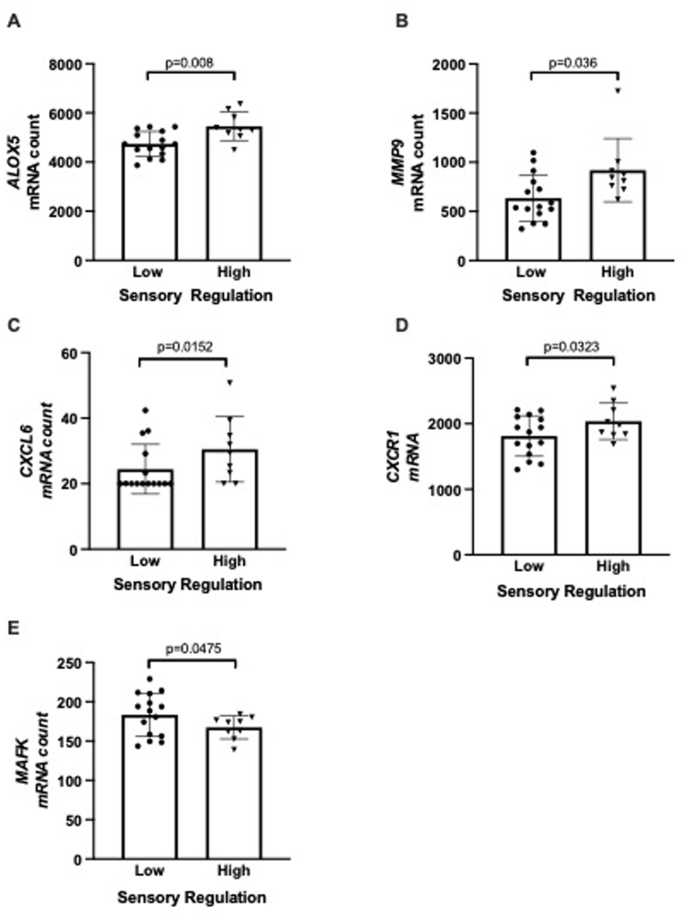
Differentially expressed genes associated with sensory regulation domain score. Graphs show individual data points, with the bar representing mean, and error bars the standard deviation.

**Fig. 3:**
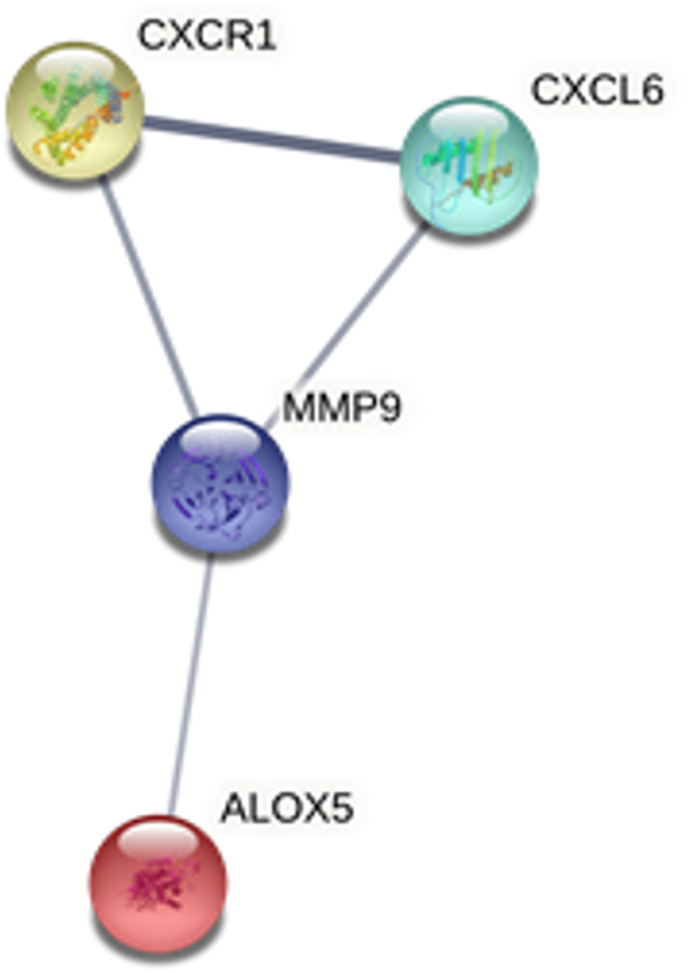
The network of protein-protein interactions between differentially expressed genes associated with sensory regulation.

Further analyses were conducted for infants meeting thresholds for autism likelihood based on their sensory regulatory domain score (“high sensory” group, n = 9, 3 male, 6 female, mean sensory regulatory domain score 23.6 ± 4.9 SD, range 15–31, versus “low sensory” group, n = 15, 10 male, 5 female, mean sensory regulatory domain score 8.1 ± 4.5 SD, range 0–14). There were no significant differences in the maternal characteristics between these groups (data not shown). Five genes were significantly differentially expressed between sensory regulatory groups (unpaired *t*-test p < 0.05, Fig. 2). These included upregulation of *ALOX5*, *CXCL6*, *CXCR1* and *MMP9*, and downregulation of *MAFK*. There were significant correlations between mRNA count and FYI sensory regulation domain score for *ALOX5* (Pearson r = −0.43, p = 0.038), and *MAFK* (Pearson r = −0.46, p = 0.022). *MAFK* mRNA count also correlated with total FYI score (Pearson r = −0.41, p = 0.046). Of these five differentially expressed genes, four are known to interact with each other in a network (*CXCR1*, *CXCL6*, *MMP-9* and *ALOX5*, Fig. 3), and all belong to the gene ontology biological process of neutrophil activation (GO:0042119, p = 0.036) and cytokine-mediated cell signalling pathway (GO:0019221, p = 0.036).

## 4 Discussion

This is the first study to examine correlations between mid-pregnancy inflammatory biomarkers and features of autism likelihood in infancy. Six inflammatory genes were up-regulated in mothers whose infants displayed a high likelihood for autism and had high scores in the social communication domain, at 12 months of age. The data also suggest that specific maternal inflammatory biomarkers may be linked to distinct autism sub-phenotypes. Mothers of infants with high sensory regulation scores on the FYI had higher levels of five genes, including *ALOX5* and *MMP9*. In addition, the sensory-regulatory domain score was significantly correlated with gene expression for *ALOX5* and *MAFK*. The differentially expressed genes associated with sensory regulation formed a network involved in neutrophil activation and cytokine-mediated cell signalling. Neutrophils are the predominant cell type at the cervicovaginal interface in early to mid-pregnancy (Mohd Zaki et al., 2022) and have been associated with poor perinatal outcomes, such as small for gestational age (Harita et al., 2012). Further, increased neutrophil activation has previously been associated with severe pre-eclampsia (Ramma et al., 2012).

A recent large study measured protein levels of 60 cytokines and growth factors in maternal mid-pregnancy blood samples from 414 autistic cases and 440 controls in the Norwegian Autism Birth Cohort (Che et al., 2022). Adjusted logistic regression models showed sex-specific associations between several cytokines in maternal blood and child autism. For example, CXCL10, one of the genes that we identified as being up-regulated in mothers whose infants were at high likelihood of autism, was higher in maternal blood of autistic males compared to male controls and mothers of autistic females with autism compared to female controls. Cord blood samples from 398 autism cases and 395 controls were also analysed; associations were not as strong as for maternal blood. The association between cord blood CXCL10 and autism likelihood was aOR 1.28 (95% CI 1.10. 1.50) for males and aOR 2.10 (95% CI 1.38, 3.20) for females.

In our study, *CYSLTR2* and *CXCL10* were significantly correlated with social communication domain score on the FYI. *CYSLTR2* is a receptor to cysteinyl leukotrienes which are leukocyte chemo-attractants which act as bronchoconstrictors, and are important mediators of asthma (Thompson et al., 2016). The gene maps to the long arm of chromosome 13 at 13q14, a region associated with asthma and other allergic diseases. The mRNA is expressed in blood eosinophils and platelets. Inhibitors of CysLTR2 and CysLTR1 are currently being evaluated in Phase II trials as asthma treatments. Further studies are required to examine the association between *CYSLTR2* and response to asthma treatment (Garcia-Menaya et al., 2019). There are no studies reporting an association between *CYSLTR2* and autism. CXCL10 is an inflammatory chemokine (also known as IP-10) that mediates immune responses by activating and recruiting leukocytes including T cells, monocytes and eosinophils. IP-10 protein levels were found to be significantly lower in the plasma of autistic children (3–6 years of age), and independently associated with social behaviours (including communication) measured by the Social Responsiveness Scale (Shen et al., 2016).

*ALOX5* and *MAFK* were significantly correlated with the sensory regulation domain score on the FYI. Like *CYSLTR2*, *ALOX5* is involved in the cysteinyl leukotriene pathway. *ALOX5* (arachidonate 5 lipoxygenase 5) is an enzyme involved in the formation of leukotrienes, and polymorphisms can result in variable production. In children with asthma, the genotype of the *ALOX5* promoter may be related to response to treatment with leukotriene receptor antagonists (Rodriguez-Martinez et al., 2020). *ALOX5* has not been previously associated with autism. *MAFK* has not been previously associated with asthma or autism.

A limitation of our study is its small sample size, which removed our ability to examine infant sex-specific effects. Replication and validation are needed in larger independent cohorts, in addition to the use of a second method (e.g., PCR) to validate mRNA expression. It would be valuable to compare the findings with a cohort of mothers without asthma, and to determine whether associations are maintained into early childhood and associated with autism diagnosis.

In conclusion, this study produces proof-of-concept evidence that in the context of maternal asthma, inflammatory gene expression in mid-pregnancy is associated with behavioural features related to later autism likelihood. Further work is needed to investigate the precise mechanisms relating maternal inflammation in pregnancy to the development of autism phenotypes in early life.

## Statements and declarations

The authors report there are no competing interests to declare.

## CRediT authorship contribution statement

**Vanessa E. Murphy:** Writing – original draft, Supervision, Project administration, Funding acquisition, Conceptualization. **Olivia M. Whalen:** Writing – review & editing, Investigation, Data curation. **Evan J. Williams:** Writing – review & editing, Methodology, Data curation. **Peter G. Gibson:** Writing – review & editing, Funding acquisition. **Linda E. Campbell:** Writing – review & editing, Supervision, Project administration, Funding acquisition, Data curation. **Frini Karayanidis:** Writing – review & editing, Supervision, Project administration. **Carly A. Mallise:** Writing – review & editing, Investigation, Data curation. **Alix Woolard:** Writing – review & editing, Investigation, Data curation. **Annelies L. Robijn:** Writing – review & editing, Formal analysis. **Joerg Mattes:** Writing – review & editing, Project administration, Funding acquisition. **Adam M. Collison:** Writing – review & editing. **Alison E. Lane:** Writing – review & editing, Supervision, Project administration, Conceptualization. **Katherine J. Baines:** Writing – review & editing, Supervision, Resources, Methodology, Formal analysis.

## Declaration of competing interest

On behalf of all listed authors of the enclosed submission, I declare that there are no known conflicts of interest, whether personal, professional, or financial.

## Data Availability

Data will be made available on request.
